# Knowledge Mapping of Human Genetic Variation in Multiomics Biomarker Translation: A Bibliometric Analysis

**DOI:** 10.1155/humu/5730260

**Published:** 2026-09-28

**Authors:** Bangshu Zhao, Wei Ran, Ning Liang

**Affiliations:** ^1^ Department of Anesthesiology, The First Affiliated Hospital of Chongqing Medical University, Chongqing, China, cqmu.edu.cn

**Keywords:** biomarker translation, biomarkers, human genetic variation, multiomics, precision medicine, targeted therapy

## Abstract

Human genetic variation and multiomics biomarker translation has emerged as a rapidly developing research field at the intersection of molecular medicine, bioinformatics, and clinical decision‐making. This study used bibliometric methods to map its global structure, intellectual base, and thematic evolution. The eligibility framework was deliberately restricted to studies in which inherited or somatic human genetic variation was substantively linked to omics‐integrated biomarker discovery, functional interpretation, clinical stratification, or therapeutic response; generic omics studies without a variation‐linked translational endpoint were excluded. Publications indexed in Scopus, Web of Science, and PubMed from 2003 to 2025 were retrieved, screened, converted into Web of Science format, and analyzed using CiteSpace, VOSviewer, and the R package bibliometrix. A total of 673 publications were included. The annual output showed sustained acceleration, indicating a transition from exploratory molecular association studies to a more mature translational framework. Research activity was concentrated in a limited number of countries and institutions, led by the United States, with China contributing substantial output but lower citation impact relative to publication volume. The institutional network was dominated by large academic medical centers, national research systems, and oncology‐oriented consortia, reflecting the infrastructural demands of biomarker translation. Journal analysis showed a dual structure in which specialized translational and omics journals accounted for much of the current output, whereas the intellectual foundations remained anchored in high‐impact journals in genetics, oncology, and clinical medicine. Keyword and overlay analyses revealed a clear thematic shift from pharmacogenetics and single‐marker discovery toward genomics‐guided, multiomics‐integrated, and clinically actionable biomarker models, with recent emphasis on liquid biopsy, heterogeneity, artificial intelligence, machine learning, and the tumor microenvironment. At the variant level, the major translational bottleneck is increasingly the functional connection between DNA‐level variation and downstream RNA, protein, pathway, and clinical phenotypes rather than variant detection alone. The literature is therefore increasingly oriented toward externally validated and computationally interpretable biomarker systems, whereas routine clinical implementation remains constrained by functional validation, assay standardization, prospective evaluation, and regulatory requirements.

## 1. Introduction

Human genetic variation is a fundamental source of interindividual differences in disease susceptibility, clinical phenotype, therapeutic response, and prognosis. In this study, “human genetic variation” includes both inherited germline and acquired somatic alterations—single‐nucleotide variants/polymorphisms, small insertions/deletions, copy‐number and structural variants—and encompasses pharmacogenomic and tumor‐genomic research when the variant itself is central to biomarker interpretation or clinical stratification [1–3]. General multiomics studies without a substantive human‐variation component were outside the target field. Advances in high‐throughput sequencing and molecular profiling have made it possible to characterize these differences at multiple biological levels, extending analysis beyond the genome to the transcriptome, proteome, metabolome, and other omics layers. This shift has changed the logic of biomarker research [4–6]. Biomarkers are no longer understood simply as isolated molecular signals; they are increasingly regarded as integrated indicators that reflect the interaction between inherited variation, somatic change, regulatory networks, and disease biology [7, 8]. In this context, the translation of multiomics biomarkers has become a central component of precision medicine.

The clinical significance of this field lies in its ability to improve diagnosis, refine risk stratification, predict treatment benefit, detect resistance, and monitor disease progression with greater biological resolution [9–11]. Genomic alterations may identify therapeutic targets or susceptibility profiles, but their predictive value is often incomplete when assessed in isolation. Transcriptomic, proteomic, and metabolomic information can complement genomic data by capturing downstream functional consequences, dynamic biological states, and microenvironmental influences [12–14]. For this reason, the integration of human genetic variation with multiomics data has become increasingly important in oncology, cardiovascular disease, neurological disorders, metabolic disease, and other complex conditions. At the same time, the field remains methodologically heterogeneous [15–17]. Differences in cohort design, specimen source, platform selection, analytical workflow, and validation strategy have produced a rapidly expanding but structurally fragmented literature.

Bibliometric analysis provides a systematic means of evaluating such a field. Unlike narrative reviews, bibliometric methods can objectively identify developmental trajectories, influential countries and institutions, source journals, collaborative patterns, core knowledge bases, and emerging themes [18–20]. This is particularly valuable in a research area shaped by both technological innovation and clinical translation, where progress is determined not only by biological discovery but also by the capacity to integrate datasets, standardize methods, and move candidate markers toward practical use [21–23]. Mapping the literature can therefore clarify how the field has evolved, where its intellectual centers are located, and which topics are defining its current direction. However, existing bibliometric studies have generally mapped precision medicine, omics, or biomarker research at a broad level and have not specifically examined how classes of human genetic variation are connected to multiomics evidence, clinical actionability, and the transition from variant detection to functional biomarker interpretation. The present study was designed to fill this field‐specific gap.

Accordingly, this study is aimed at investigating the global research landscape of human genetic variation and multiomics biomarker translation from 2003 to 2025. Using bibliometric methods based on Scopus, Web of Science, and PubMed, and visualized through CiteSpace, VOSviewer, and the R package bibliometrix, we sought to characterize publication trends, national and institutional collaboration patterns, journal structures, thematic evolution, and current research frontiers in this field. A further objective of the revised analysis was to interpret the variant‐level translational problems that are most relevant to human mutation, including functional interpretation of sequence and copy‐number variation, multiomics resolution of genotype–phenotype heterogeneity, and the clinical actionability of representative gene‐variant contexts.

## 2. Methods

### 2.1. Data Sources and Search Strategy

This bibliometric study was designed to characterize the development, intellectual structure, and translational orientation of research on human genetic variation and multiomics biomarker translation. To maximize coverage while preserving bibliographic consistency, records were retrieved from three complementary databases: Web of Science Core Collection, Scopus, and PubMed. The search window covered publications indexed from January 1, 2003, to December 31, 2025. The lower boundary of 2003 was selected to anchor the analysis in the post‐Human Genome Project transition, when a near‐complete reference genome and rapidly expanding high‐throughput genotyping and sequencing infrastructure made systematic translation of human variation increasingly feasible [24]. The retrieval strategy was not constructed as a single broad query because that approach tends to overcapture generic omics studies while underrepresenting clinically oriented biomarker work. Instead, the search framework was built around three linked conceptual domains: human genetic variation, multiomics biomarker research, and translational or clinical application.

The first domain targeted the genetic architecture of disease and included terms referring to mutation, genetic variation, genotype, polymorphism, single‐nucleotide polymorphism, genomic alteration, and related expressions. The second domain captured the multiomics dimension and incorporated terminology for genomics, transcriptomics, proteomics, metabolomics, multiomics, and integrated omics. The third domain focused on translation, including biomarker identification, diagnosis, prognosis, risk stratification, treatment selection, precision medicine, personalized medicine, targeted therapy, therapeutic response, and clinically actionable molecular profiling. Within each database, these domains were connected by Boolean operators in a concept‐block structure, with synonyms linked by “OR” and the three domains linked by “AND.” Database‐specific field restrictions were then applied. In Web of Science, the strategy was implemented at the topic level; in Scopus, the equivalent title, abstract, and keyword fields were used; and in PubMed, controlled vocabulary and title/abstract terms were combined to ensure both indexing sensitivity and semantic specificity. Pilot retrievals were used to identify recurrent sources of noise, particularly records focused on molecular mechanisms without biomarker intent, purely technical omics papers without translational endpoints, and studies in which “variation” or “omics” appeared only tangentially. The database‐specific executable syntax, field tags, date limits, and equivalent terminology are reported in Table S1. The three domains were intentionally intersected with “AND”; pilot broadening that relaxed the translational or variation requirement mainly introduced generic mechanistic or omics‐only records, so the final 673‐paper corpus should be interpreted as a high‐specificity map of the intersection rather than a census of all human‐variation or all multiomics publications.

### 2.2. Literature Screening and Eligibility Assessment

The screening process was designed to preserve conceptual precision while minimizing the loss of relevant translational studies. Eligible records included full‐length research articles and reviews when human genetic variation was an explicit exposure, stratifier, molecular determinant, or target of functional interpretation and the study addressed diagnosis, prognosis, treatment response, risk stratification, or clinically oriented biomarker development. Both germline and somatic variation were eligible; pharmacogenomics and tumor genomics were included when genotype or variant status was linked to a translational endpoint. Human relevance required human participants/biospecimens, a human disease cohort, or functional evaluation of a defined human variant with clear clinical or biomarker implications. Animal‐only, plant, microbial, and purely technical omics studies without a human‐variant translational context were excluded. No language filter was applied at database retrieval; however, records had to contain an English title and abstract sufficient for eligibility assessment and metadata harmonization. Reviews were retained because they contribute to the citation and thematic structure, whereas conference papers or abstracts, proceedings, editorials, letters, corrections, meeting notices, news items, and book chapters were excluded.

All records exported from Web of Science, Scopus, and PubMed were imported into R through the bibliometrix workflow [25]. Database‐specific exports were parsed with bibliometrix convert2df() and merged with mergeDbSources(); exact duplicate keys were DOI and PMID, followed by exact title‐year matching. Residual duplicates were assessed using normalized titles (lowercasing, punctuation/diacritic removal, and whitespace normalization) together with first author, source title, and publication year. Discordant records were retained unless identity was unambiguous. When duplicate records differed in metadata completeness, the richer bibliographic version was retained and its citation fields were preserved.

After duplicate removal, the remaining records underwent conceptual screening based on title, abstract, author keywords, and source descriptors. Ambiguous records were re‐evaluated at the full bibliographic‐record level rather than being discarded on superficial grounds. A record was excluded when the genetic component was incidental (e.g., “genomics” used only as a platform label), when no human‐variation variable or defined human variant could be identified, or when biomarker/clinical translation was absent. Records lacking sufficient metadata for bibliometric processing, especially absent title, author, source, or year fields, were also removed. This procedure yielded a final dataset of 673 publications.

### 2.3. Data Conversion, Harmonization, and Metadata Standardization

Since the three source databases use different export conventions, eligible records were harmonized into a Web of Science–compatible bibliographic structure for unified processing in CiteSpace, VOSviewer, and bibliometrix. Core fields included authors, title, source, year, affiliation, country/region, keywords, abstract, DOI/PMID, and cited references (CR). Author, journal, and affiliation variants were standardized conservatively. CR metadata were handled separately from descriptive fields. For duplicate publications, the Web of Science or Scopus record with the more complete CR field was preferentially retained. Scopus reference strings were normalized to a canonical author–year–source–volume/page–DOI representation when those elements were available. PubMed‐only records without complete CR lists were not assigned synthetic references and therefore did not contribute nonexistent outgoing edges to cocitation or CR networks, although they remained eligible for publication, collaboration, journal, and keyword analyses. Reference strings were collapsed only when DOI identity or an unambiguous combination of author, year, source, and volume/page supported equivalence; otherwise, they were kept separate. No missing CR were imputed.

Keyword cleaning unified hyphenation variants such as “multi‐omics” and “multiomics,” reconciled singular/plural forms where meaning was unchanged and corrected obvious spelling inconsistencies. Broad stop‐terms lacking topical value were removed, whereas aggressive stemming was avoided to prevent biologically distinct concepts from being collapsed. Gene symbols and mutation nomenclature were not automatically merged with common English words or gene‐family aliases unless identity was unambiguous because such normalization can create false gene‐level signals

### 2.4. Bibliometric Mapping and Knowledge‐Structure Analysis

Three software environments were used complementarily. Bibliometrix was used for descriptive bibliometrics, source/country/affiliation summaries, local citation structure, trend topics, and thematic treemaps; VOSviewer for collaboration and keyword co‐occurrence/overlay/density maps; and CiteSpace for time‐sliced country/institution networks, cited‐source mapping, keyword timelines, and dual‐map citation paths.

The principal units of analysis were annual output; country and international collaboration; institutional networks; productive and locally cited journals; cross‐disciplinary citation paths; and keyword co‐occurrence, temporal overlay, density, timeline, and trend‐topic structure.

Author and index keywords were used for conceptual mapping. Thresholds were selected iteratively to balance coverage with readability, and only stable, interpretable structures that persisted after metadata cleaning and cross‐software comparison were retained.

### 2.5. Statistical Methods

The principal indicators were annual and cumulative publication counts, yearly share and growth rate, total and local citations, source and affiliation frequency, country output, collaboration type, and keyword occurrence. Annual growth was calculated as the year‐to‐year relative change. In CiteSpace, 1‐year slices were used for 2003–2025, node selection used the g‐index (*k* = 10), Pathfinder pruning reduced redundant links, and betweenness centrality identified bridging nodes. Cluster interpretability was assessed with modularity and silhouette statistics. In VOSviewer, association‐strength normalization was used; node size represented frequency/citation magnitude and edge thickness represented link strength. Citation‐based measures were interpreted comparatively within the corpus rather than as absolute measures of scientific merit.

### 2.6. Variant‐Level Translational Synthesis

A post hoc clinical‐context synthesis was added for representative gene‐variant and mutation‐class problems linked to the dominant themes of the corpus. Since Web of Science, Scopus, and PubMed exports do not provide a standardized gene/HGVS field and gene symbols are inconsistently indexed across titles, abstracts, and keywords, this component was not used to generate pseudoprecise numerical gene rankings. Instead, clinically established or emerging gene‐variant contexts and mutation classes were interpreted using consensus/guideline and functional‐interpretation literature [1–3, 26–31]. This clinical‐context synthesis used literature published within the bibliometric window and did not alter bibliometric counts or networks.

## 3. Results

### 3.1. Publication Volume Analysis

As shown in Figure 1 and Table 1, the literature on human genetic variation and multiomics biomarker translation exhibited a clear long‐term expansion from 2003 to 2025, with annual output increasing from 1 publication in 2003 to 146 in 2025, and cumulative output reaching 673 publications overall. The growth trajectory was not linear, but it was structurally upward across the entire observation period. During the initial phase (2003–2010), publication volume remained low and fluctuating, with annual output generally below 10 papers, indicating that the field was still conceptually dispersed and methodologically immature. At that stage, research on human genetic variation, omics technologies, and biomarker development often progressed in parallel rather than as an integrated translational framework. The subsequent period (2011–2018) showed a steady expansion, with annual publications rising from 10 to 39, suggesting that the field began to consolidate as high‐throughput sequencing, omics profiling, and computational integration became more accessible and clinically relevant. From 2019 onward, growth accelerated further, despite short‐term fluctuations in 2021 and 2022, and annual output rose sharply to 66 in 2023, 73 in 2024, and 146 in 2025; notably, the 2025 publications alone accounted for 21.69% of the total corpus, indicating that the field has entered a stage of rapid knowledge accumulation. This pattern reflects several characteristics of the field: strong dependence on technological innovation, increasing integration of genomics with other omics layers, and a growing translational demand for biomarkers capable of improving diagnosis, prognosis, patient stratification, and therapeutic decision‐making. The intermittent declines observed in several years are therefore better understood as short‐term adjustments within a broader expansionary trend rather than as evidence of stagnation. Publication dynamics indicate that this field has moved from exploratory development to accelerated translational maturation.

**Figure 1:**
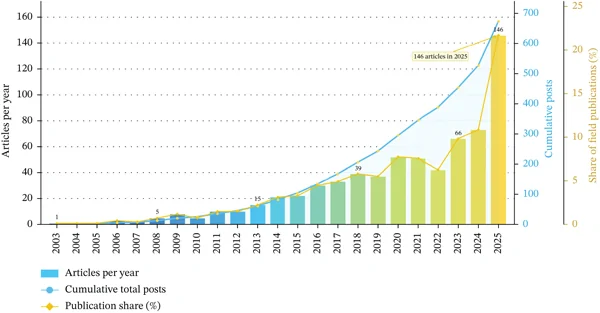
Annual publication output, cumulative growth, and publication share in research on human genetic variation and multiomics biomarker translation (2003–2025).

**Table 1:** Number of publications and trends.

Year	Articles	Total number of posts	Percentage of publications in that year relative to the total number of publications in the field	Growth rate
2003	1	1	0.15%	NaN
2004	1	2	0.15%	0.00%
2005	1	3	0.15%	0.00%
2006	3	6	0.45%	200.00%
2007	2	8	0.30%	−33.33%
2008	5	13	0.74%	150.00%
2009	8	21	1.19%	60.00%
2010	5	26	0.74%	−37.50%
2011	10	36	1.49%	100.00%
2012	10	46	1.49%	0.00%
2013	15	61	2.23%	50.00%
2014	21	82	3.12%	40.00%
2015	22	104	3.27%	4.76%
2016	30	134	4.46%	36.36%
2017	33	167	4.90%	10.00%
2018	39	206	5.79%	18.18%
2019	37	243	5.50%	−5.13%
2020	52	295	7.73%	40.54%
2021	51	346	7.58%	−1.92%
2022	42	388	6.24%	−17.65%
2023	66	454	9.81%	57.14%
2024	73	527	10.85%	10.61%
2025	146	673	21.69%	100.00%

### 3.2. Country Analysis

The national distribution of research on human genetic variation and multiomics biomarker translation shows a markedly uneven structure, characterized by concentration in a small group of scientifically mature countries and by collaboration patterns organized around a transatlantic–East Asian axis. According to Table 2, the United States ranked first in both publication output (245) and total citations (16,712), indicating not only the largest research volume but also the strongest overall academic influence. China ranked second in publications (132) but only seventh in citations (2216), whereas England produced 55 papers yet ranked second in citations (8650), and Denmark entered the citation top three despite not appearing among the 10 most productive countries. This discrepancy suggests that productivity and influence are not fully aligned in this field; countries with fewer papers may exert disproportionate impact when they contribute foundational methods, cohort resources, or highly visible translational studies. The network structures further clarify this pattern. In the CiteSpace map, the United States occupies the dominant hub position and maintains broad links with Europe and Asia, whereas England, Germany, Italy, and China also form prominent nodes in the international network (Figure 2). The VOSviewer clustering map shows that collaboration is not random but organized into regional and functional groupings, with the United States and China anchoring a major Asian‐Pacific cluster, England and Germany connecting a dense European core, and Spain, Portugal, Brazil, and Mexico forming a smaller but coherent collaboration group (Figure 3). The global collaboration map confirms that cross‐national cooperation is concentrated in North America, Western Europe, and East Asia, with relatively limited participation from Africa and much of Latin America, except for a few active countries such as South Africa and Brazil (Figure 4). Figure S1 further indicates that the United States and China rely heavily on single‐country publications in absolute terms, although the United States also has the largest volume of multiple‐country publications, reflecting greater capacity to lead international collaboration.

**Table 2:** Top 10 countries by number of publications and citations.

Publication rankings	Country	Publication	Citation rankings	Country	Citations
1	United States	245	1	United States	16,712
2	China	132	2	England	8650
3	Italy	57	3	Denmark	6487
4	England	55	4	Canada	2885
5	Germany	53	5	Spain	2434
6	Canada	38	6	Germany	2410
7	France	37	7	China	2216
8	Spain	37	8	France	1967
9	India	28	9	Italy	1188
10	South Korea	28	10	Switzerland	1000

**Figure 2:**
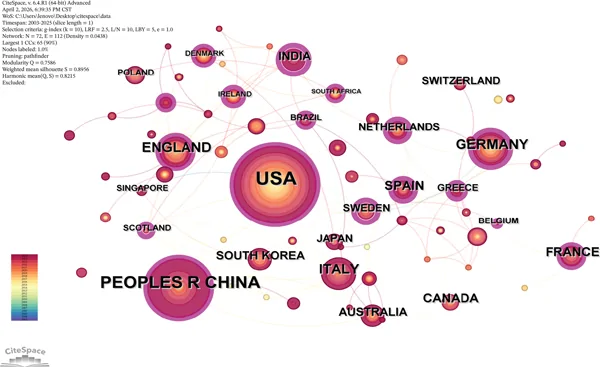
Country collaboration network in research on human genetic variation and multiomics biomarker translation.

**Figure 3:**
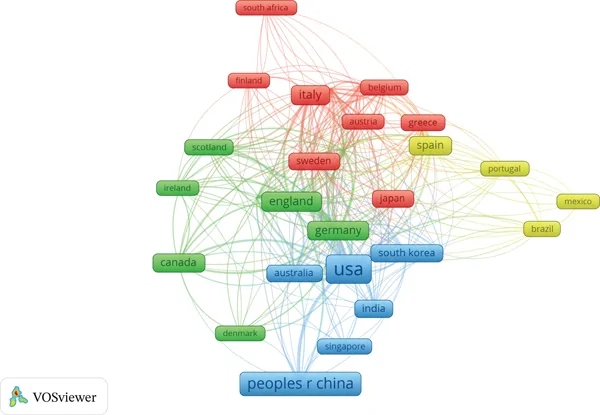
Clustered coauthorship network of countries/regions in research on human genetic variation and multiomics biomarker translation.

**Figure 4:**
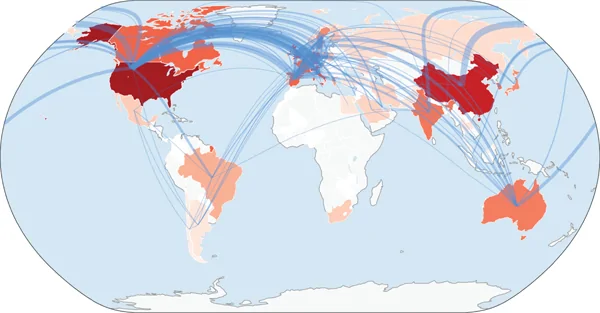
Global collaboration map of countries/regions in research on human genetic variation and multiomics biomarker translation.

Analytically, the output–citation asymmetry should not be read as a direct quality ranking. Citation age, journal mix, international coauthorship, participation in reusable cohorts or standards, and network centrality can all change aggregate citation impact; these factors are considered explicitly in the Section 4.

### 3.3. Journal Analysis

Journal distribution in this field shows a clear dual structure, with publication activity concentrated in specialized open‐access molecular and translational journals, whereas intellectual influence is anchored in a smaller set of high‐impact general science, clinical medicine, and oncology journals. The source overlay map indicates that recent publishing activity has been especially prominent in Cancers, International Journal of Molecular Sciences, Biomolecules, Diagnostics, Frontiers in Oncology, and Scientific Reports, whereas earlier or comparatively more established nodes such as Pharmacogenomics, Genome Medicine, and Journal of Translational Medicine occupy cooler temporal positions, suggesting that the field has shifted from early pharmacogenomic and proteomic translation toward broader multiomics integration and clinically oriented biomarker deployment (Figure S2). This pattern is consistent with the source productivity ranking, in which Cancers (26 papers), International Journal of Molecular Sciences (25 papers), and OMICS: A Journal of Integrative Biology (19 papers) were the most productive outlets, followed by Frontiers in Oncology and Genes (11 papers each) (Figure S3). These journals are well aligned with the field′s knowledge requirements: They accommodate interdisciplinary work combining genomics, transcriptomics, proteomics, bioinformatics, and translational oncology, and they also provide relatively rapid dissemination for emerging biomarker studies. However, the cocited journal network reveals a different hierarchy of influence. The most central and frequently cocited journals include Nature, New England Journal of Medicine, Journal of Clinical Oncology, Clinical Cancer Research, Nature Genetics, Nature Communications, Cell, Science, Cancer Research, and Proceedings of the National Academy of Sciences of the United States of America (Figure S4), a pattern reproduced in the local citation ranking, where Nature (1863 local citations), New England Journal of Medicine (1545), and Journal of Clinical Oncology (1302) clearly dominate (Figure S5). These results indicate that the field publishes much of its incremental output in specialized translational journals but builds its conceptual and evidentiary foundation on highly authoritative journals in genomics, oncology, and clinical medicine, reflecting the fact that biomarker translation depends simultaneously on methodological innovation, biological discovery, and clinical validation.

### 3.4. Analysis of Institutions

The institutional structure of research on human genetic variation and multiomics biomarker translation is characterized by a high degree of concentration in large academic medical centers, national biomedical research systems, and specialized cancer networks, with collaboration organized around several densely connected transnational hubs. In the institutional network, the most prominent nodes include Harvard University, the University of California System, the National Institutes of Health (NIH), Harvard Medical School, Harvard University Medical Affiliates, INSERM, the University of Toronto, Memorial Sloan Kettering Cancer Center, and the University of Texas System, indicating that the field is led not by isolated laboratories but by integrated research ecosystems combining basic science, clinical cohorts, and translational infrastructure (Figure S6). This pattern is reinforced by the publication ranking of affiliations, in which Harvard University (69 articles) clearly leads, followed by the University of California System (46), UNICANCER (44), the University of Toronto (44), Harvard University Medical Affiliates (41), Helmholtz Association (38), and INSERM (37) (Figure S7). The coexistence of universities, national research agencies, and hospital‐based consortia among the leading institutions suggests that this field depends on organizational forms capable of linking genomics, multiomics profiling, clinical annotation, and validation pipelines within a single collaborative framework. The density map further shows that activity is clustered in several institutional cores rather than evenly distributed, with particularly strong hotspots around the Toronto–McGill–Baylor axis, the Harvard medical ecosystem, and a distinct Chinese cluster centered on Fudan University and China Medical University, whereas additional nodes such as Karolinska Institutet, the National University of Singapore, and the University of Ljubljana occupy more peripheral but still visible positions (Figure S8). These findings indicate that the field has a structurally centralized yet internationally networked configuration.

The concentration of the network around academic medical ecosystems is analytically important because the limiting resource is not a single omics platform but the ability to connect variant annotation, biospecimens, multilayer assays, longitudinal phenotypes, and independent validation within the same translational pipeline.

### 3.5. Analysis of Dual‐Map Overlay

The dual‐map overlay indicates that research on human genetic variation and multiomics biomarker translation is organized around a distinctly translational citation structure in which knowledge is produced mainly in journals positioned within the domains of molecular biology, immunology, and clinical medicine, and is cited primarily from journals concentrated in molecular biology, genetics, and health‐related medicine (Figure S9). The thickest citation trajectories run from the left‐side clusters labeled “Molecular, Biology, Immunology” and “Medicine, Medical, Clinical” toward the right‐side clusters “Molecular, Biology, Genetics” and “Health, Nursing, Medicine,” showing that the field is not confined to a single disciplinary core but instead links mechanistic discovery with clinical interpretation and application. This pattern reflects the internal logic of biomarker translation: Studies of genetic variation and multiomics signatures usually originate from basic and translational bioscience, but their ultimate scientific value depends on validation against clinical phenotypes, disease classification, prognosis, and treatment response. At the same time, additional citation paths extend toward areas such as systems computing and computer science, as well as chemistry, materials, and physics, suggesting that the field also depends on computational modeling, algorithmic integration, sequencing technologies, mass spectrometry, and other analytical platforms that make multilayer molecular profiling possible. The overlay therefore reveals three structural characteristics of the field. First, it is biologically grounded, with molecular genetics as its principal knowledge base. Second, it is clinically oriented, because much of the citation traffic converges on medical and health‐related domains rather than remaining within basic biology alone. Third, it is methodologically composite, drawing on informatics and analytical sciences to convert high‐dimensional omics data into clinically interpretable biomarkers. The significance of this pattern is that progress in the field depends less on isolated disciplinary advances than on sustained integration across laboratory biology, computational analysis, and clinical research, which is consistent with the broader movement from omics discovery toward precision medicine and actionable biomarker development.

### 3.6. Analysis of Keywords and Trends in the Field

The keyword structure indicates that this field is organized around a translational core linking molecular variation, omics integration, biomarker development, and precision oncology. In the CiteSpace timeline, early high‐frequency terms were centered on relatively discrete molecular and pharmacogenetic topics, including gefitinib, prostate‐specific antigen, pharmacogenetics, single nucleotide polymorphism, gene‐expression profiles, and trial‐related terms, showing that the field initially advanced through candidate markers, drug‐response studies, and single‐layer molecular assays rather than through fully integrated multiomics models (Figure S10). This developmental logic is also visible in the co‐occurrence network, where biomarkers, mutations, gene, association, and risk form one major cluster, whereas precision medicine, personalized medicine, cancer, targeted therapy, and immunotherapy define a second cluster, and genomics, proteomics, transcriptomics, gene‐expression, and multiomics define a third cluster that bridges molecular profiling with clinical use (Figure S11). The field therefore has three clear characteristics: It is strongly disease‐oriented, with oncology occupying the dominant application space; it is methodologically integrative, with genomics, proteomics, transcriptomics, and multiomics functioning as linked rather than isolated analytical layers; and it is clinically stratified, as reflected by the prominence of survival, prognosis, heterogeneity, diagnosis, liquid biopsy, and circulating tumor DNA. The trend‐topic map further shows a chronological transition from pharmacogenetics, genotype, and sequencing‐related topics in the earlier stage, to genomics, proteomics, mutations, biomarkers, cancer, and precision medicine in the intermediate stage, and more recently to multiomics, machine learning, artificial intelligence, tumor microenvironment, and site‐specific applications such as gastric cancer (Figure S12). This shift is convincing in light of the field′s underlying technical and clinical drivers: Once sequencing and proteomic platforms became more scalable, the major bottleneck moved from detecting variation to integrating heterogeneous molecular signals into robust diagnostic, prognostic, and therapeutic models. The treemap confirms this hierarchy, with precision medicine (192), biomarkers (162), genomics (135), mutations (123), and personalized medicine (112) occupying the largest conceptual areas, followed by proteomics, biomarker, metabolomics, cancer, and expression (Figure S13). The keyword evolution suggests that the field has moved from single‐marker and single‐omics discovery toward integrative, model‐based, and clinically actionable biomarker translation, with oncology remaining the principal testing ground because of its molecular heterogeneity, strong therapeutic stratification needs, and the relative availability of tissue, blood‐based, and longitudinal outcome data.

The recent AI/machine‐learning signal is therefore not interpreted here as a generic biomedical fashion. In the context of this corpus, it accompanies the transition toward cross‐omics data fusion, subtype classification, treatment‐response prediction, resistance modeling, and reduction of high‐dimensional molecular data to clinically testable signatures; the specific computational implications are developed in Section 4.3.

### 3.7. Gene‐ and Variant‐Level Translational Interpretation

Bibliographic metadata support robust thematic mapping but not a reliable HGVS‐level frequency ranking, because gene symbols and mutation nomenclature are incompletely and inconsistently represented in titles, abstracts, and keywords. To avoid false precision, the revised manuscript therefore adds a clinically oriented gene‐variant extension rather than unsupported frequency counts. Table S2 focuses on EGFR activating/resistance variants, KRAS G12C, BRCA1/2 pathogenic variants, and TP53 hotspot/Y220C alterations as representative contexts that directly connect human variation to biomarker actionability. EGFR, KRAS G12C, and BRCA1/2 already guide approved therapy in defined disease settings, whereas most TP53 alterations remain context‐dependent biomarkers and allele‐specific p53 reactivation is still emerging [27–30].

At the mutation‐class level, the recurrent translational problem is functional interpretation: DNA detection alone does not establish whether a missense, truncating, splice‐altering, copy‐number, or structural variant changes transcript processing, protein abundance/function, pathway state, or drug sensitivity. This distinction is central to multiomics translation because RNA and protein layers can supply orthogonal evidence for variant pathogenicity or biological activity [1–3, 26, 31].

## 4. Discussion

### 4.1. Countries/Institutions and Their Cooperation

The growth of this literature is most informative when viewed as a change in the unit of translation: The field has moved from asking whether a variant is associated with a phenotype toward asking whether the variant produces a reproducible molecular consequence and retains predictive value across cohorts, assays, and clinical settings. This shift makes harmonized phenotyping, longitudinal sampling, and external validation as important as sequencing capacity itself.

The geographical and institutional distributions indicate a pronounced concentration of activity in countries and centers with strong biomedical infrastructure. The United States occupies the dominant position in output, citations, and network connectivity, whereas China has emerged as a major contributor in publication volume but has lower aggregate citation impact. This publication–citation gap should not be interpreted as evidence of inferior originality from the present data alone. Aggregate citations are sensitive to publication age, journal distribution, international coauthorship, and whether a research system contributes reusable cohorts, reference resources, methodological standards, or multicenter trials. A plausible explanation is that part of China′s recent growth is concentrated in disease‐specific and single‐center translational studies, whereas many historically highly cited standards and multinational resources remain anchored in US/European networks; however, this bibliometric design cannot causally separate these mechanisms. The more actionable implication is to strengthen internationally harmonized, deeply phenotyped cohorts, external validation, and shared data infrastructures rather than to prioritize publication volume itself.

At the institutional level, the dominance of large academic medical centers, national research systems, and cancer‐focused consortia is structurally meaningful. Human genetic variation and multiomics biomarker translation is not a field that can be advanced by isolated laboratory work alone. It depends on repeated interaction between molecular scientists, clinicians, pathologists, bioinformaticians, statisticians, and trial investigators [32–34]. Institutions such as Harvard, the University of California System, NIH‐linked centers, INSERM, Toronto‐based networks, and major cancer centers appear prominent because they embody this organizational integration. Their importance lies not merely in productivity, but in their ability to connect discovery platforms with validation cohorts and clinical implementation pathways. This also explains why collaboration networks are dense in North America and Western Europe and increasingly strong in East Asia: These regions have invested most heavily in infrastructure capable of sustaining translational omics research [35–37]. For the field to advance further, collaboration must become not only broader, but also more functionally integrated, with stronger links between high‐throughput discovery, external validation, and real‐world clinical deployment. Network centrality in this setting is therefore best understood as a marker of infrastructural integration: Institutions that can connect variant discovery to pathology, multiomics assays, bioinformatics, longitudinal outcomes, and prospective trials are more likely to generate results that become reusable across diseases and countries.

### 4.2. Journals and the Knowledge Base of the Field

The journal pattern suggests a field with a dual publishing ecology. Much of the current output is concentrated in specialized or interdisciplinary journals that accept translational genomics, multiomics, oncology, and biomarker studies. By contrast, the most influential intellectual foundations are concentrated in a smaller group of highly cited journals in general medicine, oncology, molecular biology, and genetics. This distinction is important [38–40]. It indicates that the field generates its incremental advances in journals designed for rapid communication of specialized findings, but continues to derive its conceptual authority from journals that publish decisive methodological, mechanistic, or clinically practice‐shaping work. The divergence between productive and cocited journals also indicates an evidentiary asymmetry: Discovery reports can proliferate faster than consensus standards, external validation, and practice‐changing trials, so publication productivity should not be equated with translational maturity.

Specialized journals are well suited to disease‐specific biomarker panels and proof‐of‐concept omics studies, whereas the most cited sources tend to establish mechanistic frameworks, analytical standards, or clinical utility [41–46]. For human genetic variation, this distinction matters because a statistically associated marker becomes clinically durable only after variant interpretation, functional validation, assay reproducibility, and decision‐level utility have converged.

The dual‐map overlay reinforces this interpretation. The dominant citation paths run from molecular biology, immunology, and clinical medicine toward molecular genetics and health‐related medicine, showing that the field is constructed through constant movement between mechanistic insight and clinical application. This is consistent with the nature of multiomics biomarker translation. A biomarker is rarely valuable because it is statistically associated with disease alone. Its durability depends on biological interpretability, technical reproducibility, and clinical relevance [47–49]. The current publishing structure therefore reflects a field that is scientifically hybrid: It remains rooted in molecular sciences, but its trajectory is increasingly determined by whether molecular findings can survive the demands of clinical validation.

### 4.3. Research Hotspots and Frontiers

The thematic evolution of this field reveals a clear shift from single‐layer molecular association toward integrated and clinically oriented biomarker systems. Earlier work was organized around pharmacogenetics, single nucleotide polymorphisms, gene‐expression profiles, targeted therapy markers, and candidate variation analysis. These topics were important because they established the principle that inherited and somatic variation could stratify patients, predict drug response, and inform prognosis. However, they also reflected the limits of the earlier phase of the field. Most studies relied on one molecular layer, relatively narrow disease contexts, or single‐marker reasoning.

The more recent keyword structure shows that this framework has been superseded by a broader model centered on precision medicine, biomarkers, genomics, proteomics, transcriptomics, metabolomics, multiomics, liquid biopsy, circulating tumor DNA, heterogeneity, prognosis, and increasingly artificial intelligence and machine learning. This transition is not superficial. It marks a conceptual shift in how translational value is understood. The central challenge is no longer whether molecular variation matters, but how different layers of molecular information can be integrated into models that are robust enough for clinical use. Tumor heterogeneity, temporal evolution, treatment resistance, and microenvironmental effects all weaken the performance of single biomarkers [50–52]. Multiomics approaches have become prominent because they are better suited to capturing the biological complexity that underlies clinical variability.

Oncology is the dominant application space in this field, and for understandable reasons. Tumors are genetically heterogeneous, clinically stratified, and heavily dependent on treatment selection. They also provide a relatively rich environment for biomarker research because tissue, plasma, longitudinal outcomes, and therapy‐response data are often available in ways that are less common in many nonneoplastic diseases [53–56]. As a result, cancer has become the principal testing ground for multiomics translation, especially in areas such as targeted therapy, immunotherapy, acquired resistance, and noninvasive monitoring. However, the prominence of oncology should not be interpreted as a limitation of scope alone. It also indicates where the translational burden is highest and where the clinical return on biomarker refinement is most immediate.

The AI/machine‐learning hotspot has a specific role in this field rather than representing a generic biomedical trend. Current methods are used principally to (i) compress and integrate high‐dimensional, heterogeneous omics layers in “many‐features/few‐samples” settings; (ii) classify molecular subtypes and predict prognosis or drug response from joint genomic, transcriptomic, proteomic, and clinical features; and (iii) prioritize variant‐associated molecular states for validation [57, 58]. In inherited common disease, machine learning also contributes to polygenic risk‐score construction and calibration, but that is a narrower branch of the corpus and remains constrained by ancestry portability and uncertain incremental clinical utility [59]. Thus, the relevant frontier is not AI itself, but whether computational integration can convert cross‐modal molecular variation into reproducible, interpretable, externally validated decisions. Many proposed signatures are statistically impressive but clinically fragile. Reproducibility across cohorts, platforms, specimen types, and analytic pipelines remains uneven [60, 61]. Biomarker models are often built in retrospective datasets, optimized on high‐dimensional inputs, and validated in settings that do not reflect routine clinical practice [62, 63].

### 4.4. Variant‐Level Bottlenecks and Clinical Actionability

Different variation classes create different translational bottlenecks. Common SNPs can be statistically robust yet functionally indirect because linkage disequilibrium obscures causal alleles and effect sizes vary across ancestry; rare missense variants generate a large burden of variants of uncertain significance; splice‐site and deep‐intronic variants may require RNA evidence; copy‐number/structural variants require breakpoint, dosage, mosaicism, and platform‐aware interpretation; and somatic variants must be interpreted in the context of tumor purity, subclonality, comutation, and temporal evolution [1–3, 26, 31]. Multiomics can close part of this gap by linking a DNA event to allele‐specific expression or splicing, protein abundance and pathway activity, and ultimately phenotype, thereby distinguishing a detected variant from a biologically active biomarker.

Clinical actionability is likewise allele‐, disease‐, and context‐specific. EGFR activating variants in nonsmall‐cell lung cancer are embedded in guideline‐based tyrosine–kinase‐inhibitor pathways [27]; germline BRCA1/2 pathogenic variants can determine PARP‐inhibitor eligibility in metastatic breast cancer [28]; and KRAS G12C has become directly targetable in previously treated nonsmall‐cell lung cancer [29]. TP53 illustrates the opposite problem: It is biologically prominent and frequently comutated, but most TP53 alterations do not yet confer a uniform gene‐wide treatment indication [64–66]. Allele‐specific p53 Y220C reactivation with rezatapopt provides a therapeutic proof of concept and is under clinical evaluation, emphasizing that actionability must be assigned at the variant rather than gene‐name level [30].

### 4.5. Future Directions and Implications

The next stage should prioritize multicenter validation for biomarker classes that are especially vulnerable to sampling and model drift: multigene/multiomics prognostic signatures, liquid‐biopsy models for minimal residual disease or acquired resistance, and AI‐derived treatment‐response classifiers. In oncology, lung, breast/ovarian, colorectal, and gastric cancers are immediate test beds because the corpus is densely organized around targeted therapy, ctDNA/liquid biopsy, heterogeneity, and site‐specific multiomics applications. In human genetics outside oncology, transcriptomic or proteomic confirmation of splice effects and variants of uncertain significance is an equally important translational route. External validation must move closer to prospective and multicenter designs. Biomarker translation requires evidence that remains stable across institutions, populations, specimen processing protocols, and sequencing platforms. Specific bottlenecks that should be prespecified include preanalytical timing, variant nomenclature, cross‐platform normalization, batch correction, missing‐data handling, temporal sampling, calibration, ancestry portability, and clinically meaningful decision thresholds.

These considerations suggest that the maturation of the field will depend on a change in emphasis. The next stage is not merely the expansion of omics discovery, but the disciplined conversion of molecular complexity into clinically usable evidence. That requires shared standards in sample collection, preprocessing, feature selection, model construction, outcome definition, and reporting. It also requires closer integration between computational development and clinical decision‐making. In this sense, the future of human genetic variation and multiomics biomarker translation lies less in discovering ever more markers than in establishing which markers are portable, mechanistically grounded, and decision‐relevant. Regulatory qualification, cost‐effectiveness, laboratory turnaround time, and integration into existing clinical workflows should be evaluated alongside predictive performance; without these steps, a high‐performing multiomics model remains a research result rather than a deployable biomarker.

### 4.6. Strengths and Limitations

This study provides a comprehensive overview of the developmental trajectory, collaborative structure, publication ecology, and thematic evolution of research on human genetic variation and multiomics biomarker translation. By integrating multiple bibliometric tools and combining three major databases, it captures both macro‐level trends and internal knowledge structures more effectively than a single‐source analysis. It also clarifies how the field has shifted from pharmacogenetic association and single‐marker discovery toward multiomics integration, precision medicine, and computationally assisted biomarker modeling. Several limitations should be acknowledged. Bibliometric results are influenced by database coverage, indexing rules, citation lag, and source standardization procedures. Older publications have had more time to accumulate citations, whereas recent but potentially important topics may appear underrepresented in citation‐based analyses. Converting records from different databases into a unified format improves comparability but can reduce some metadata granularity and may not fully eliminate institutional or author‐name variation. In addition, bibliometric analysis can identify influential themes and structural relationships, but it cannot directly determine the methodological quality, biological validity, or clinical utility of individual biomarker studies. These limitations do not weaken the overall pattern observed here, but they should be considered when interpreting fine‐grained rankings and emerging trends. The deliberately intersective eligibility criteria also explain the relatively modest corpus size: The 673 records represent a high‐specificity set in which human variation, omics evidence, and translational biomarker intent co‐occur, not an exhaustive census of all human genetic variation or all multiomics biomarker research. This specificity can miss disease‐specific papers that do not use generic variation/omics terminology, which is why the complete search syntax is provided in Table S1 for future sensitivity testing. In addition, PubMed‐only records without complete CR lists could not contribute absent outgoing citation edges; no reference metadata were imputed. Finally, the merged bibliographic exports do not contain a standardized gene/HGVS field; therefore, a quantitative gene/mutation‐frequency ranking would risk nomenclature‐driven misclassification. The gene‐variant extension in Table S2 is therefore explicitly interpretive rather than a substitute for full‐text gene‐frequency mining.

## 5. Conclusion

Human genetic variation and multiomics biomarker translation has evolved into a rapidly expanding and increasingly integrated research field with clear translational relevance to precision medicine. This bibliometric study delineates its global growth pattern, leading contributors, institutional and journal structures, and the thematic progression from pharmacogenetics and single‐marker discovery toward genomics‐guided, multiomics‐based, and clinically actionable biomarker models. The field is anchored in oncology, but it extends beyond cancer to the broader problem of linking molecular heterogeneity with diagnosis, prognosis, treatment selection, and disease monitoring. Current research is shaped by the convergence of genomics, transcriptomics, proteomics, metabolomics, liquid biopsy, and computational modeling, whereas the main challenge has shifted from biomarker discovery to biomarker validation and implementation. The literature is increasingly oriented toward clinical deployment, but actual implementation remains a major challenge. The next stage should emphasize multicenter prospective validation, harmonized preanalytical and analytical workflows, ancestrally and clinically diverse populations, allele‐specific functional interpretation, and interpretable machine‐learning models linked to longitudinal clinical data. Regulatory qualification, clinical utility, and workflow feasibility must accompany predictive performance. Progress will therefore depend not on generating more molecular features alone, but on establishing which variant‐linked multiomics biomarkers are functionally grounded, reproducible, and sufficiently validated to support real‐world decisions.

## Author Contributions

Bangshu Zhao: conceptualization, methodology, validation, formal analysis, investigation, resources, data curation, writing—original draft, writing—review and editing, project administration. Wei Ran: conceptualization, methodology, validation, formal analysis, investigation, resources, data curation, writing—original draft, writing—review and editing, project administration. Ning Liang: conceptualization, methodology, validation, formal analysis, investigation, resources, data curation, writing—original draft, writing—review and editing, project administration.

## Funding

No funding was received for this manuscript.

## Ethics Statement

This study has been reviewed by the Ethics Committee of the First Affiliated Hospital of Chongqing Medical University, which determined that it is a bibliometric study involving neither patients nor animals and therefore does not require approval to proceed.

## Conflicts of Interest

The authors declare no conflicts of interest.

## Supporting Information

Additional supporting information can be found online in the Supporting Information section.

## Supporting information


**Supporting Information 1** Figure S1: Corresponding author′s countries: single‐country and multiple‐country publications.

**Supporting Information 2** Figure S2: Overlay visualization of active journals in research on human genetic variation and multiomics biomarker translation.

**Supporting Information 3** Figure S3: Most relevant publication sources in research on human genetic variation and multiomics biomarker translation.

**Supporting Information 4** Figure S4: Cocited journal network in research on human genetic variation and multiomics biomarker translation.

**Supporting Information 5** Figure S5: Most locally cited sources in research on human genetic variation and multiomics biomarker translation.

**Supporting Information 6** Figure S6: Institutional collaboration network in research on human genetic variation and multiomics biomarker translation.

**Supporting Information 7** Figure S7: Most relevant research affiliations in human genetic variation and multiomics biomarker translation.

**Supporting Information 8** Figure S8: Density visualization of research institutions in human genetic variation and multiomics biomarker translation.

**Supporting Information 9** Figure S9: Dual‐map overlay of journals in research on human genetic variation and multiomics biomarker translation.

**Supporting Information 10** Figure S10: Keyword time‐zone map of research on human genetic variation and multiomics biomarker translation.

**Supporting Information 11** Figure S11: Keyword co‐occurrence network in research on human genetic variation and multiomics biomarker translation.

**Supporting Information 12** Figure S12: Trend topics in research on human genetic variation and multiomics biomarker translation.

**Supporting Information 13** Figure S13: Treemap of high‐frequency keywords in research on human genetic variation and multiomics biomarker translation.

**Supporting Information 14** Table S1: Reproducible database‐specific search syntax corresponding to the final three‐block retrieval framework.

**Supporting Information 15** Table S2: Variant‐centered clinical interpretation added.

## Data Availability

The data that support the findings of this study are available from the corresponding author upon reasonable request.
