# Supplementary material for: Knowledge Mapping of Human Genetic Variation in Multiomics Biomarker Translation: A Bibliometric Analysis
Source: Hum Mutat. 2026 Sep 28;2026:5730260. doi: 10.1155/humu/5730260 (PMC13618366; doi:10.1155/humu/5730260)
Supplement: Supplementary file 1 [file HUMU-2026-5730260-s012.docx]

**Supplementary Table S1. Reproducible database-specific search syntax corresponding to the final three-block retrieval framework.**

| **Database** | **Executable search syntax** | **Limits/fields** |
| --- | --- | --- |
| Web of Science Core Collection | TS=(("human genetic variation" OR mutation* OR genotype* OR polymorphism* OR SNP* OR "single nucleotide polymorphism*" OR "single nucleotide variant*" OR "genomic alteration*" OR "germline variant*" OR "somatic variant*" OR "copy number variation*" OR CNV* OR "structural variant*" OR pharmacogenom*) AND (multiomic* OR "multi-omic*" OR genomics OR transcriptomic* OR proteomic* OR metabolomic* OR epigenomic* OR "integrated omic*" OR "omics integration") AND (biomarker* OR diagnos* OR prognos* OR "risk stratification" OR "treatment selection" OR "therapeutic response" OR "drug response" OR "precision medicine" OR "personalized medicine" OR "targeted therapy" OR "clinical actionab*" OR "molecular profil*")) AND PY=(2003-2025) | Topic field; publication years 2003-2025. Human relevance and document type were applied during screening. |
| Scopus | TITLE-ABS-KEY(("human genetic variation" OR mutation* OR genotype* OR polymorphism* OR SNP* OR "single nucleotide polymorphism*" OR "single nucleotide variant*" OR "genomic alteration*" OR "germline variant*" OR "somatic variant*" OR "copy number variation*" OR CNV* OR "structural variant*" OR pharmacogenom*) AND (multiomic* OR "multi-omic*" OR genomics OR transcriptomic* OR proteomic* OR metabolomic* OR epigenomic* OR "integrated omic*" OR "omics integration") AND (biomarker* OR diagnos* OR prognos* OR "risk stratification" OR "treatment selection" OR "therapeutic response" OR "drug response" OR "precision medicine" OR "personalized medicine" OR "targeted therapy" OR "clinical actionab*" OR "molecular profil*")) AND PUBYEAR > 2002 AND PUBYEAR < 2026 | Title/abstract/keyword fields; human relevance and document type were applied during screening. |
| PubMed | (("Genetic Variation"[Mesh] OR "Mutation"[Mesh] OR "Polymorphism, Genetic"[Mesh] OR "Pharmacogenetics"[Mesh] OR "genetic variation"[Title/Abstract] OR mutation*[Title/Abstract] OR genotype*[Title/Abstract] OR polymorphism*[Title/Abstract] OR SNP*[Title/Abstract] OR "single nucleotide polymorphism"[Title/Abstract] OR "single nucleotide variant"[Title/Abstract] OR "genomic alteration"[Title/Abstract] OR "germline variant"[Title/Abstract] OR "somatic variant"[Title/Abstract] OR "copy number variation"[Title/Abstract] OR "structural variant"[Title/Abstract] OR pharmacogenom*[Title/Abstract]) AND ("Genomics"[Mesh] OR "Transcriptome"[Mesh] OR "Proteomics"[Mesh] OR "Metabolomics"[Mesh] OR multiomic*[Title/Abstract] OR "multi-omic"[Title/Abstract] OR genomics[Title/Abstract] OR transcriptomic*[Title/Abstract] OR proteomic*[Title/Abstract] OR metabolomic*[Title/Abstract] OR epigenomic*[Title/Abstract] OR "integrated omics"[Title/Abstract] OR "omics integration"[Title/Abstract]) AND ("Biomarkers"[Mesh] OR "Precision Medicine"[Mesh] OR biomarker*[Title/Abstract] OR diagnos*[Title/Abstract] OR prognos*[Title/Abstract] OR "risk stratification"[Title/Abstract] OR "treatment selection"[Title/Abstract] OR "therapeutic response"[Title/Abstract] OR "drug response"[Title/Abstract] OR "precision medicine"[Title/Abstract] OR "personalized medicine"[Title/Abstract] OR "targeted therapy"[Title/Abstract] OR "clinical actionability"[Title/Abstract] OR "molecular profiling"[Title/Abstract])) AND ("2003/01/01"[Date - Publication] : "2025/12/31"[Date - Publication]) | Controlled vocabulary plus title/abstract terms; publication date 2003/01/01-2025/12/31. Human relevance and document type were applied during screening to avoid over-restrictive indexing filters. |
