# Supplementary material for: Knowledge Mapping of Human Genetic Variation in Multiomics Biomarker Translation: A Bibliometric Analysis
Source: Hum Mutat. 2026 Sep 28;2026:5730260. doi: 10.1155/humu/5730260 (PMC13618366; doi:10.1155/humu/5730260)
Supplement: Supplementary file 2 [file HUMU-2026-5730260-s015.docx]

**Supplementary Table S2. Variant-centered clinical interpretation added.**

| **Gene/variant context** | **Established or emerging clinical role** | **What multiomics contributes** | **Key translational limitation** |
| --- | --- | --- | --- |
| EGFR activating/resistance variants | Actionable in EGFR-mutant NSCLC through guideline-based TKI pathways [61]. | ctDNA and transcript/protein states can track clonal evolution, resistance, and pathway reactivation. | Allele-specific resistance, co-mutations, tissue/ctDNA discordance, and temporal heterogeneity. |
| BRCA1/2 pathogenic variants | Can determine PARP-inhibitor eligibility in defined breast-cancer settings [62]. | Genomic scars, transcript/protein evidence, and broader homologous-recombination states can refine biological context. | A pathogenic gene variant is not interchangeable with a validated multiomics HRD biomarker; assay definitions and disease context matter. |
| KRAS G12C | Directly targetable in previously treated KRAS G12C-mutant NSCLC [63]. | Co-mutation, expression, signaling, and immune-context layers can help explain variable response and resistance. | Actionability is allele-specific; other KRAS alleles and co-alterations have different therapeutic implications. |
| TP53 hotspot/Y220C alterations | Most TP53 alterations remain context-dependent; Y220C has allele-specific reactivation evidence with a clinical-stage agent [64]. | Transcript/protein/pathway readouts help distinguish loss-of-function, dominant-negative, gain-of-function, and co-mutation effects. | High gene-level prevalence does not imply uniform therapeutic actionability; interpretation must be variant-specific. |
| Missense, truncating, splice-altering, CNV/SV classes | Clinical significance ranges from benign to pathogenic/actionable and requires standardized variant interpretation [53-55]. | RNA-seq can demonstrate aberrant splicing/expression; proteomic and pathway readouts can provide orthogonal functional evidence [57,65]. | VUS burden, tissue-specific expression, breakpoint/dosage effects, mosaicism, and assay/platform dependence. |
